# VNN3 is a potential novel biomarker for predicting prognosis in clear cell renal cell carcinoma

**DOI:** 10.1080/19768354.2019.1583126

**Published:** 2019-03-01

**Authors:** Mihyang Ha, Hoim Jeong, Jong Seong Roh, Beomgu Lee, Dongjun Lee, Myoung-Eun Han, Sae-Ock Oh, Dong Hyun Sohn, Yun Hak Kim

**Affiliations:** a Department of Anatomy, Pusan National University School of Medicine, Yangsan, Republic of Korea; b Department of Microbiology and Immunology, Pusan National University School of Medicine, Yangsan, Republic of Korea; c Department of Convergence Medical Science, Pusan National University School of Medicine, Yangsan, Republic of Korea; d Department of Biomedical Informatics, Pusan National University School of Medicine, Yangsan, Republic of Korea; e Biomedical Research Institute, Pusan National University Hospital, Busan, Republic of Korea

**Keywords:** VNN3, biomarker, clear cell renal cell carcinoma, TCGA, ICGC

## Abstract

Although pathological observations provide approximate prognoses, it is difficult to achieve prognosis in patients with existing prognostic factors. Therefore, it is very important to find appropriate biomarkers to achieve accurate cancer prognosis. Renal cell carcinoma (RCC) has several subtypes, the discrimination of which is crucial for proper treatment. Here, we present a novel biomarker, VNN3, which is used to prognose clear cell renal cell carcinoma (ccRCC), the most common and aggressive subtype of kidney cancer. Patient information analyzed in our study was extracted from The Cancer Genome Atlas (TCGA) and International Cancer Genome Consortium (ICGC) cohorts. VNN3 expression was considerably higher in stages III and IV than in stages I and II. Moreover, Kaplan–Meier curves associated high VNN3 expression with poor prognoses (TCGA, *p* < .0001; ICGC, *p* = .00076), confirming that ccRCC prognosis can be predicted via VNN3 expression patterns. Consistent with all patient results, the prognosis of patients with higher VNN3 expression was worse in both low stage (I and II) and high stage (III and IV) (TCGA, *p* < 0.0001 in stage I and II; ICGC, *p* = 0.028 in stage I and II; TCGA, *p* = 0.005 in stage III and IV). Area under the curve and receiver operating characteristic curves supported our results that highlighted VNN3 expression as a suitable ccRCC biomarker. Multivariate analysis also verified the prognostic performance of VNN3 expression (TCGA, *p* < .001; ICGC, *p* = .017). Altogether, we suggest that VNN3 is applicable as a new biomarker to establish prognosis in patients with ccRCC.

## Introduction

Renal cell carcinoma (RCC) is the 6th most common cancer in men worldwide, accounting for about 5% of all oncology-diagnosed cases, and the 10th most common cancer in women with a rate of about 3% (Siegel et al. 2018). RCC has several histological subtypes, including clear cell RCC (ccRCC), papillary RCC (pRCC), and chromophobe RCC (chRCC), of which ccRCC is the most common subtype, accounting for 70–80% of all incidences (Truong and Shen 2011; Srigley et al. 2013). Patients with ccRCC also have five-year disease-specific survival rates as high as about 69%, and tend to have a worse prognosis than patients with other RCC subtypes (Cheville et al. 2003; Gudbjartsson et al. 2005; Patard et al. 2005). The prognosis of RCC patients has traditionally depended on clinically relevant techniques such as histological stage and grade (Amin et al. 2002; Gudbjartsson et al. 2005; McLaughlin et al. 2006). However, these classic prognostic variables may be affected by inter-observer variability and may not fully account for individual tumor biology in RCC cases. To overcome these shortcomings, biomarkers have recently been introduced to achieve prognosis (Brannon et al. 2010; Finley et al. 2011).

Biomarkers identify a broad subcategory of medical symptoms, which helps in accurately reproducing the signs of a patient’s medical conditions even in *ex vivo* circumstances (Strimbu and Tavel 2010). In the last two decades of cancer research, the application of biomarkers in cancer clinical trials has resulted in significant progress in this area by widening our across-the-spectrum understanding of early and late cancers (Reid and Yasko 2012). A new molecular classification system using biomarkers needs to be considered to complement the classical cancer classification system. As additional clinical indices are discovered based on the prognostic and predictive factors of cancer via these biomarkers, they should be applied to the staging guidelines in order to develop new cancer treatment strategies (Amin et al. 2017).

VNN3 is a member of the vanin family that shares a very high sequential similarity with the other members and encodes for an ectoenzyme with pantetheinase activity. The vanin gene family is known to be involved in oxidative stress and inflammation (Mariani and Roncucci 2017). Here, we suggest VNN3 as an important prognostic biomarker for ccRCC using two cohorts, The Cancer Genome Atlas (TCGA) (Cerami et al. 2012; Cancer Genome Atlas Research Network et al. 2013) and International Cancer Genome Consortium (ICGC) (International Cancer Genome Consortium et al. 2010).

## Materials and methods

### Patient data accession and characteristics

Clinical and genomic data for TCGA and ICGC cohorts were downloaded from the ICGC data portal (https://dcc.icgc.org) in March 2018. Patients with insufficient clinicopathological data were excluded. Overall, 58% of patients in TCGA (*n* = 262) and 66% of patients in ICGC (*n* = 60) were classified as low stage (I and II). Approximately 57% of the patients in both cohorts were male. Detailed patient characteristics are summarized in Table 1. Statistical analysis was performed using the R statistical software (The R Foundation for Statistical Computing, 2018).

**Table 1.** Patient characteristics (TCGA and ICGC cohorts).

		TCGA (*n* = 446)	ICGC (*n* = 91)
Stage	I	216	48
	II	46	12
	III	111	13
	IV	71	9
	Not available	2	9
Grade	I	9	–
	II	189	–
	III	175	–
	IV	68	–
	Not available	5	–
Gender	Male	290	52
	Female	156	39
Age (mean ± sd)		60.62 ± 12.80	60.47 ± 10.03

### Wilcoxon signed-rank test

Due to abnormal distribution, the differences in the VNN3 expression values between low stage (I and II) and high stage (III and IV) in both cohorts were identified via the Wilcoxon signed-rank test using the ‘coin’ package.

### Survival analysis

We performed survival analysis to predict the overall survival (OS) of TCGA and ICGC cohorts. In the survival curves (Kaplan–Meier), we determined a cut-off value that had the maximal Uno’s C-index using a previously described five-fold cross-validation method (Cho et al. 2018; Ha et al. 2018). To evaluate the discriminatory power, we used a log-rank test with the Uno’s C-index in a time-dependent area under the curve (AUC) analysis and the AUC values in receiver operating characteristic (ROC) curves at a two-year period, as described previously (Han et al. 2018; Goh et al. 2019). These values were obtained using the R packages, ‘survival’ and ‘survAUC’. We also used univariate and multivariate Cox regression to compare the prognostic effect of VNN3 expression as a categorical factor and other clinical variables. All statistical analyses were performed using R.

## Results

### Patient information from TCGA and ICGC

We analyzed a total of 446 patients from TCGA and 91 patients from ICGC for this study (Table 1). In TCGA cohort, 290 were male and 156 were female, and in the ICGC cohort, 52 were male and 39 were female. The number of patients for each stage in TCGA cohort were 216 (stage I), 46 (stage II), 111 (stage III), and 71 (stage IV). The number of patients for each stage in the ICGC cohort were 48 (stage I), 12 (stage II), 13 (stage III), and 9 (stage IV). The patients who did not have stage information (two patients in TCGA and nine patients in ICGC) were not included in the analysis based on cancer stage, but included in the analysis containing all patients.

### VNN3 expression values based on ccRCC stage

**Figure 1.**
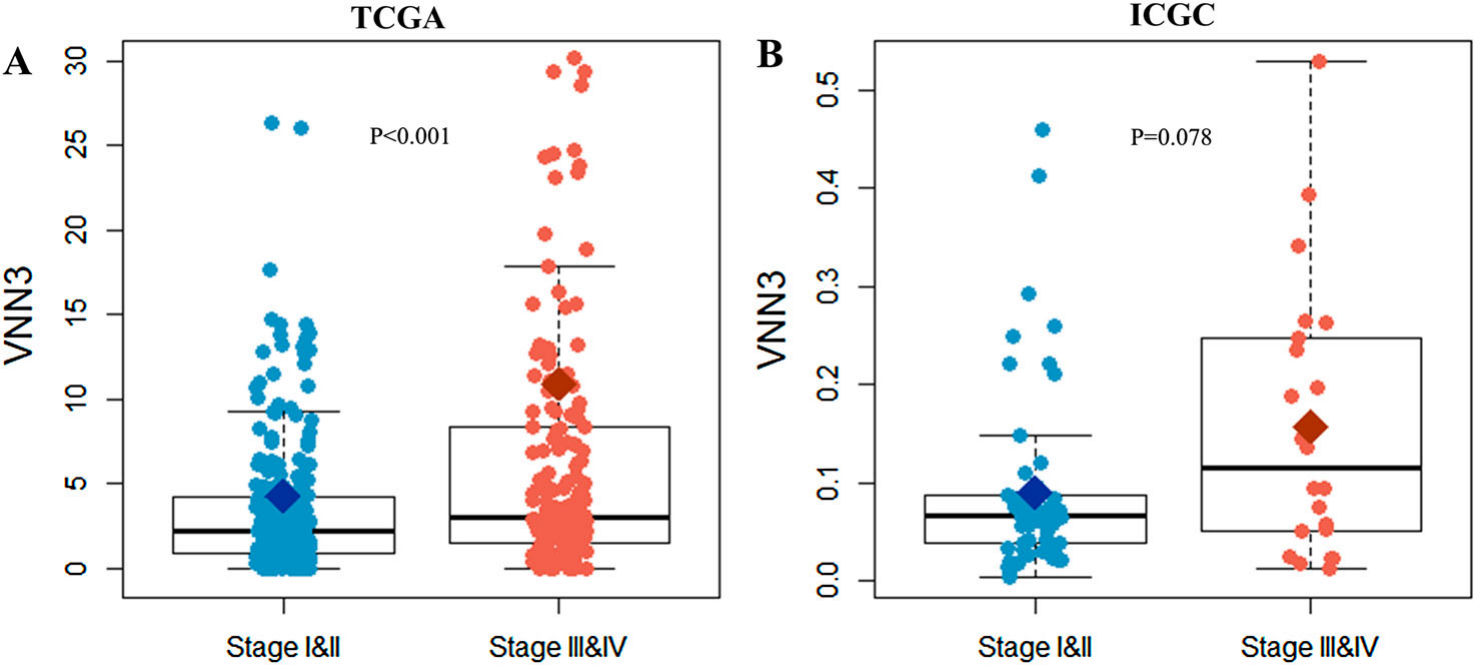
Comparison of VNN3 gene expression. VNN3 gene expression was compared between stages I and II and III and IV in ccRCC patients from TCGA (A) and ICGC (B) cohorts. TCGA, The Cancer Genome Atlas; ICGC, International Cancer Genome Consortium.

To compare the expression value of VNN3 in patients with ccRCC, we performed a Wilcoxon signed-rank test on TCGA and ICGC data (Figure 1 and Table 2). We found that VNN3 expression in stages III and IV was significantly higher than that in stages I and II in TCGA cohort (*p* < .001; Figure 1(A)), but not in the ICGC cohort (Figure 1(B)). The VNN3 expression values ranged from 0 to 431.034 in TCGA with a 1st quarter value of 1.022, median value of 2.644, and 3rd quarter value of 5.140. ICGC cohort showed a range of 0.003931–0.530031 with a 1st quarter value of 0.038821, median value of 0.070559, and 3rd quarter value of 0.110752 for VNN3 expression. For further analysis, we set the cut-off to 3.8153 for TCGA and 0.071537 for ICGC (Table 2).

**Table 2.** VNN3 expression values for TCGA and ICGC cohorts.

		TCGA	ICGC
VNN3 expression value	Minimum	0	0.003931
	1st quarter	1.022	0.038821
	Median	2.644	0.070559
	3rd quarter	5.140	0.110752
	Maximum	431.034	0.530031
	Cut-off	3.8153 (26^th^)	0.071537 (47^th^)

### VNN3 expression as a prognostic value in ccRCC

**Figure 2.**
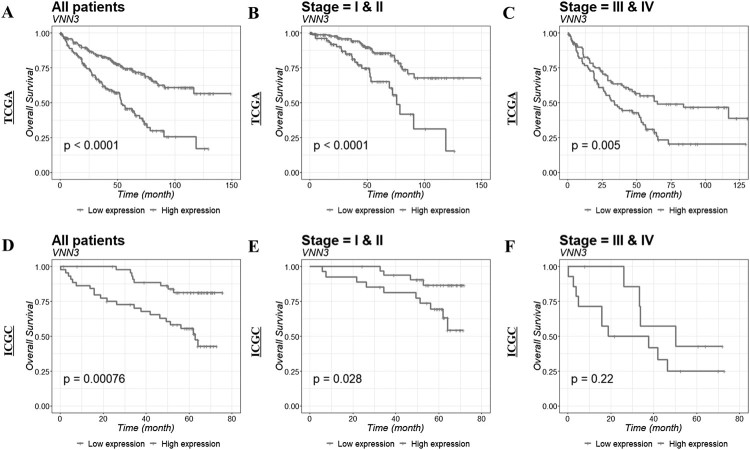
Kaplan–Meier survival analysis of ccRCC patients with respect to VNN3 gene expression. Survival analysis was performed in ccRCC patients from TCGA (A, B, C) and ICGC (D, E, F) cohorts. Survival analysis was also compared based on the following subgroups: all patients (A and D), stages I and II (B and E), and stages III and IV (C and F). *p*-Value was calculated using log-rank test and is provided at the bottom left of each dataset.

We evaluated the possibility of VNN3 as a prognostic marker in ccRCC by analyzing Kaplan–Meier curves that compared the relationship between VNN3 expression level and patient survival. Notably, patients with low VNN3 expression showed a higher survival rate than those with high VNN3 expression in TCGA (*p* < .0001; Figure 2(A)) and ICGC (*p* = .00076; Figure 2(D)). Furthermore, we examined the differences in survival rates based on the stage and VNN3 expression. We confirmed that survival rates were higher in patients with low VNN3 expression when compared to high VNN3 expression in stages I and II of TCGA (*p* < .0001; Figure 2(B)) and ICGC (*p* = .028; Figure 2(E)) cohorts. However, survival rates were higher in patients with low VNN3 expression in stages III and IV of TCGA cohort (*p* = .005; Figure 2(C)), but not in stages III and IV of the ICGC cohort (*p* = .22; Figure 2(F)). We also verified the possibility of VNN3 as a prognostic marker using multivariate analysis. As shown in Table 3, VNN3 had prognostic significance in both TCGA (*p* < .001) and ICGC (*p* = .017) cohorts.

**Table 3.** Univariate and multivariate analysis of overall survival in each cohort.

Parameters	Univariate analysis				Multivariate analysis			
	*p*	HR	95 Cl		*p*	HR	95 Cl	
TCGA								
Age	<.001	1.033	1.018	1.047	<.001	1.030	1.015	1.046
Stage (I, II vs. III, IV)	<.001	3.478	2.474	4.888	<.001	2.794	1.947	4.009
Gender (female vs. male)	.333	0.850	0.612	1.181	.492	1.130	0.797	1.609
Grade (I, II vs. III, IV)	<.001	2.247	1.572	3.212	.143	1.331	0.908	1.950
VNN3	<.001	2.570	1.860	3.551	<.001	2.057	1.469	2.881
ICGC								
Age	.109	1.031	0.993	1.071	.269	1.023	0.982	1.066
Stage (I, II vs. III, IV)	<.001	4.796	2.264	10.16	<.001	4.265	1.950	9.327
Gender (female vs. male)	.863	1.066	0.517	2.194	.858	0.931	0.428	2.028
VNN3	.002	3.671	1.632	8.259	.017	2.760	1.201	6.341

### Verification of VNN3 expression as a new biomarker for ccRCC

**Figure 3.**
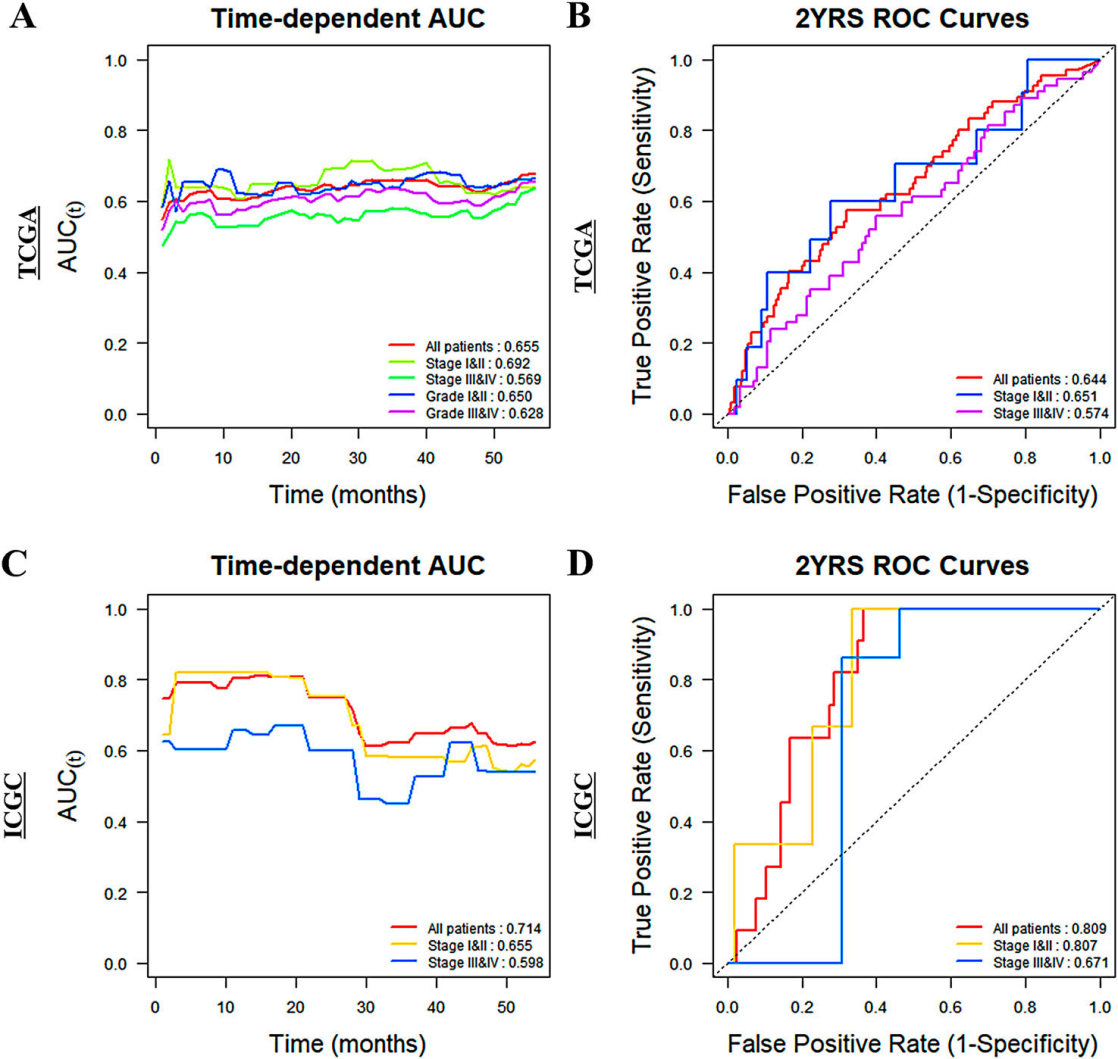
Time-dependent AUC analysis and ROC curves at 2 years with respect to VNN3 gene expression in TCGA and ICGC cohorts. (A) Time-dependent AUC in TCGA (red, all patients; light green, stages I and II; green, stages III and IV; blue, grades I and II; purple, grades III and IV). (B) ROC curve at 2 years in TCGA (red, all patients; blue, stages I and II; purple, stages III and IV). (C) Time-dependent AUC in ICGC (red, all patients; yellow, stages I and II; blue, stages III and IV). (D) ROC curve at 2 years in ICGC (red, all patients; yellow, stages I and II; blue, stages III and IV). C-index values for A and C, and AUC values at 2 years for B and D are provided at the bottom right of each dataset.

We determined Uno’s C-index values in the time-dependent AUC analysis and AUC values in the ROC curves to estimate the performance of VNN3 expression as a new biomarker for ccRCC (Figure 3). In the time-dependent AUC analysis of TCGA cohort (Figure 3(A)), the highest C-index value was observed in stages I and II (0.692), followed by all patients (0.655), grades I and II (0.650), grades III and IV (0.628), and stage III and IV (0.569). The highest C-index value for the ICGC cohort was observed in all patients (0.714; Figure 3(C)). The 2-year ROC curves for both TCGA and ICGC showed high values in all the patient groups, highlighting VNN3 as a good prognostic biomarker for the relatively early stages of ccRCC (Figure 3(B,D)).

## Discussion

In the present study, we investigated the performance of VNN3 as a prognostic factor in ccRCC. Its prognostic value was assessed via information obtained from a total of 537 patients from TCGA and ICGC databases, which were used to interactively explore multidimensional cancer genome studies. VNN3 expression levels were observed to increase during the late stages of ccRCC in TCGA, which suggested that elevated VNN3 levels adversely affected ccRCC progression. Although the VNN3 expression in the ICGC cohort showed a tendency similar to TCGA cohort, the *p*-value obtained was not significant (*p* = .078; Figure 1(B)). These results could probably be attributed to the small number of patients obtained from ICGC (*n* = 91) when compared to TCGA (*n* = 446; Table 1). The lack of patients in ICGC also appeared to have affected the results of VNN3 expression in stages III and IV (Figure 2(F)), which only had 22 patients (Table 1).

VNN3 is a member of the vanin gene family that consists of VNN1, VNN2, and VNN3. This gene family encodes pantetheinase, which hydrolyzes pantetheine into pantothenic acid (vitamin B5) and the antioxidant, cysteamine (Mariani and Roncucci 2017). A specific role of the vanin gene family in inflammation and diseases was previously suggested. VNN1 deficiency showed a decrease in intestinal inflammation and protection from colitis (Martin et al. 2004; Berruyer et al. 2006), VNN2 was reported to regulate adhesion and migration of activated neutrophils (Suzuki et al. 1999), and VNN3 was highly expressed in inflamed human skin conditions such as psoriasis and atopic dermatitis lesions (Jansen et al. 2009). Recent studies also showed the involvement of VNN3 in 1,4-benzoquinone-induced cell proliferation by regulating the expression of cell proliferation-related genes such as KLF15 and NOTCH1 (Sun et al. 2018). Moreover, VNN3 expression was also shown to increase after hepatitis C virus infection or epidermal growth factor receptor activation in hepatoma cells (Benkheil et al. 2018). These results suggest the possible role of VNN3 in cancer development.

Unlike the other vanins, VNN3 appears to encode a secreted protein (Mariani and Roncucci 2017). Plasma VNN3 has also been reported to be associated with gastrointestinal acute graft-versus-host disease in mice (Wang et al. 2018). In this study, we found that high VNN3 expression correlated to a bad ccRCC prognosis based on the mRNA expression database. Therefore, further studies are needed to clarify if VNN3 induces ccRCC and if the plasma level of VNN3 correlates with ccRCC.
